# Curdlan, zymosan and a yeast-derived β-glucan reshape tumor-associated macrophages into producers of inflammatory chemo-attractants

**DOI:** 10.1007/s00262-020-02707-4

**Published:** 2020-08-29

**Authors:** Priscilla de Graaff, Cor Berrevoets, Christiane Rӧsch, Henk A. Schols, Kees Verhoef, Harry J. Wichers, Reno Debets, Coen Govers

**Affiliations:** 1 Laboratory of Tumor Immunology, Department of Medical Oncology, Erasmus MC-Cancer Institute, Rotterdam, The Netherlands; 2 Wageningen Food and Biobased Research, Wageningen UR, Wageningen, The Netherlands; 3 Laboratory of Food Chemistry, Wageningen University, Wageningen, The Netherlands; 4 Department of General Surgery, Erasmus MC-Cancer Institute, Rotterdam, The Netherlands

**Keywords:** Beta-glucans, Macrophages, Chemokines, Chemo-attractants

## Abstract

Anti-cancer T-cell responses are often halted due to the immune-suppressive micro-environment, in part related to tumor-associated macrophages. In the current study, we assessed indigestible β-glucans (oatβG, curdlan, grifolan, schizophyllan, lentinan, yeast whole glucan particles (yWGP), zymosan and two additional yeast-derived β-glucans a and b) for their physicochemical properties as well as their effects on the plasticity of human monocyte-derived macrophages that were polarized with IL-4 to immune-suppressive macrophages. Beta-glucans were LPS/LTA free, and tested for solubility, molecular masses, protein and monosaccharide contents. Curdlan, yeast-b and zymosan re-polarized M(IL-4) macrophages towards an M1-like phenotype, in particular showing enhanced gene expression of *CCR7*, *ICAM1* and *CD80*, and secretion of TNF-α and IL-6. Notably, differential gene expression, pathway analysis as well as protein expressions demonstrated that M(IL-4) macrophages treated with curdlan, yeast-b or zymosan demonstrated enhanced production of chemo-attractants, such as CCL3, CCL4, and CXCL8, which contribute to recruitment of monocytes and neutrophils. The secretion of chemo-attractants was confirmed when using patient-derived melanoma-infiltrating immune cells. Taken together, the bacterial-derived curdlan as well as the yeast-derived β-glucans yeast-b and zymosan have the unique ability to preferentially skew macrophages towards a chemo-attractant-producing phenotype that may aid in anti-cancer immune responses.

**Electronic supplementary material:**

The online version of this article (10.1007/s00262-020-02707-4) contains supplementary material, which is available to authorized users.

## Introduction

The efficacy of anti-tumor immune therapies is challenged by the immune-suppressive micro-environment. Novel strategies are being developed to specifically target suppressive immune cells in tumors [1]. Examples of such immune-suppressive cell populations include tumor-associated neutrophils, regulatory T cells (Tregs), myeloid derived suppressor cells (MDSCs) and tumor-associated macrophages (TAMs) [2]. TAMs are an abundant immune cell population in many types of solid tumors, such as those derived from breast, ovarian, bladder and skin and can be subdivided into macrophages that either promote or suppress immune activation [3, 4]. Macrophages can acquire a continuum of phenotypes and functions that is generally captured by two hallmark phenotypes termed classically activated macrophages or M1 and alternatively activated macrophages or M2 [5]. Interestingly, T helper type 1 (Th1) cells produce anti-tumoral IFN-γ that gives rise to M1 macrophages [6], whereas Th2 cells or Tregs secrete pro-tumoral cytokines, such as IL-4 and IL-10 [7], that give rise to M2 macrophages or TAMs. TAMs are predominantly present at later stages of tumor progression, and closely resemble M2 macrophages. These cells often interact with CD8 T cells at tumor margins and prevent CD8 T cells to migrate and invade the tumor [8]. In addition, TAMs may result in enhanced expression of programmed cell death ligand 1 (PD-L1), PD-L2, CD80 and CD86 by tumor cells, which upon interaction with cognate receptors can dampen CD8 T-cell responses [9, 10]. Following recognition of their roles in immune evasion, TAMs are now considered potential targets for cancer therapeutic approaches [11]. Currently, major anti-TAM treatments used in cancer patients generally target macrophage recruitment through CSF1R inhibitors or anti-CLL2 neutralizing antibodies or target macrophage reprogramming through PI3Kγ inhibitors [12].

Beta-glucans comprise a class of indigestible polysaccharides that have been reported to support anti-tumor effects [2]. For example, adjuvant use of β-glucans in treating hepatocellular carcinoma, gastric cancer and colorectal cancer with chemo-or radiotherapy increased 5-year survival up to 15% and reduces recurrence by as much as 43% [13–15]. Moreover, it has been demonstrated that orally administered yeast-derived β-glucan supports the anti-tumor activity of adoptively transferred T cells and re-directs MDSC into an M1-like phenotype thereby facilitating recruitment of dendritic cells and macrophages towards tumors [16, 17]. The immunomodulatory activity of β-glucans is considered to be mediated via their recognition by pattern recognition receptors (PRRs) of which many are expressed by macrophages, such as dectin-1, lactosylceramide receptor (LacCer), scavenger receptor, mannose receptor and complement receptor 3 (CR3) [2]. A single PRR may recognize several types of β-glucans, and alternatively, a single β-glucan can be bound by several receptors. For instance, dectin-1 binds the β-glucans curdlan, zymosan, scleroglucan, lentinan, WGP and schizophyllan [2], whereas curdlan and zymosan are bound by dectin-1 as well as Toll-like receptor 2 (TLR2) [2] The differential (and cumulative) signaling from PRRs that are ligated by a β-glucan will ultimately determine the downstream cellular responses, which is nicely illustrated by Noss and colleagues, who exposed whole blood to a large variety of β-glucans and demonstrated unique profiles of cytokine production [18].

Receptor binding of β-glucans in general, and therefore the immunomodulatory activity of β-glucans, depends heavily on their physicochemical structure [19]. Beta-glucans consist of β-1,3-linked glucose units with either β-1,4 or β-1,6 branching, the exact structure being related to its source. For example, curdlan, a bacterium derived β-glucan is linear, non-branched and consists of only β-1,3-linked glucose units. OatβG, a cereal β-glucan, is linear, non-branched and consists of β-1,4-linked glucose units connected to each other by β-1,3 linkages, while beta-glucans derived from yeast or fungi are non-linear and consist of β-1,3-linked glucose units with β-1,6-linked glucose branches that vary in length or contain branch-to-branch structures [20, 21].

Over the last decades many immune therapy trials have been conducted in melanoma, and objective response rates ranged from approximately 15% for cytokine administrations to up to 50% for adoptive transfer of expanded tumor-infiltrating lymphocytes. Despite these successes, only a minor fraction of patients (i.e., 5–20%) demonstrates durable clinical responses [22], which, given their high abundance in the microenvironments of melanoma [23], may be related to TAMs. In this study, we investigated nine β-glucans from various sources for their physicochemical properties, an aspect that is underappreciated in many studies, as well as their impact towards the phenotype and function of in vitro IL-4-polarized or patient melanoma-derived macrophages using biochemical tests, gene expression and pathway analysis as well as protein expression assays. Our study showed that stimulation with β-glucans derived from *Alcaligenes faecalis* (i.e., curdlan) or yeast (i.e., yeast-b and zymosan) skew TAMs to a more pro-inflammatory phenotype, characterized by a unique and significant gene expression and secretion of chemo-attractants.

## Materials and methods

### Beta-glucan preparations

We used an oat-derived β-glucan termed oatβG (Megazyme, Bray, Ireland), a bacteria-derived (*Alcaligenes faecaeli*) β-glucan termed curdlan (Megazyme), three fungi-derived β-glucans termed grifolan (*grifola frondosa* (Hangzhou New Asia International Co., Ltd, Hangzhou, China))*,* schizophyllan (*schizophyllum commune* (Invivogen, Toulouse, France)) and lentinan (*lentinula edodus* [24]), and four yeast-derived (*saccharomyces cerevisiae*) β-glucans termed yeast whole glucan particle (yWGP (Invivogen)), zymosan (Invivogen), yeast-a (Megazyme), and yeast-b (Immitec, Tonsberg, Norway). All nine β-glucans were available as dry powder. Stock solutions were made of 1 mg/ml dry powder suspended in RPMI 1640 (Gibco, Life Technologies, Bleiswijk, The Netherlands) with 10% fetal bovine serum (FBS; Gibco) and 1% penicillin and streptomycin (Sigma-Aldrich, Zwijndrecht, The Netherlands). OatβG was boiled in distilled water for 10 min before processing, lentinan was stirred O/N at 37 °C and zymosan was stirred for 2 h at 37 °C before these preparations were tested.

### Presence and removal of LPS and LTA from β-glucan preparations

HEK-Blue hTLR2 and hTLR4 (Invivogen) cells were used to detect and quantify presence of lipopolysaccharides (LPS) or lipoteichoic acid (LTA) in β-glucan preparations. HEK cells were cultured in T75 flasks in DMEM with 10% FBS and 1% antibiotics. LPS/LTA assays were performed as described elsewhere [25]. In short, HEK cells were exposed to 0.001–1000 EU/ml LPS (*E. coli* 0111:B4, Sigma–Aldrich), 1–25 µg/ml LTA (*Staphylococcus Aureus*, Sigma–Aldrich) or β-glucans at a final concentration of 500 µg/ml for 24 h. Secreted embryonic alkaline phosphatase (SEAP), a surrogate marker for LPS/LTA, was quantified by mixing 20 μl of supernatant with 180 μl of Quanti-Blue™ in a flat bottom 96-well plate. The plate was incubated for 2 h at 37 °C and absorption was determined by spectrophotometry at 655 nm (TECAN, Giessen, The Netherlands). In addition, LPS was also quantified by the EndoZyme® test kit (Hyglos GmbH, Bernried am Starnberger See, Germany); spike controls were taken alongside for all β-glucan preparations. Beta-glucans that were contaminated with LPS (> 0.002 ng/ml), LTA (> 1 µg/ml) or both were treated as previously described [25]. In brief, β-glucan preparations were treated with alkaline-ethanol at 56 °C for 5 h and were lyophilized following neutralization with HCl. Finally, absolute ethanol was added and samples were stored O/N at 4 °C. Molecular mass distribution was analyzed before and after LPS/LTA removals using HPSEC. Beta-glucan preparations were recovered by centrifugation for 20 min at 3,320* g* and washed three times with 60% ethanol prior to their use.

### Molar mass, solubility, protein and saccharide content of β-glucan preparations

Beta-glucan preparations were exposed to high pressure size exclusion chromatography (HPSEC) analysis to determine molar masses and solubility. In short, preparations were suspended in water (10 mg/ml) and heated to 80 °C for 10 min. Samples were centrifuged (10 min, RT, 18,000* g*) and supernatants were analyzed using an Ultimate 3000 HPLC (Dionex, Sunnyvale, CA, USA) equipped with a Shodex RI-101 refractive index detector (Showa Denko, Tokyo, Japan). For separations, three TSK-Gel columns (Tosoh Bioscience, Tokyo, Japan) connected in series (4000–3000–2500 SuperAW; 150 × 6 mm) were preceded by a TSK Super AW-L guard column (35 × 4.6 mm). 10 µl of samples was injected and eluted with 0.2 M NaNO_3_ at 55 °C at a flow rate of 0.6 ml/min. Pullulan molecular-mass standards from 0.2 to 780 kDa (Polymer Laboratories, Palo Alto, CA, USA) were used for calibration. Solubility of β-glucan preparations is assessed according to areas under the HPSEC curves in comparison to oatβG, which has a solubility of 100%. HPSEC is based on the soluble part of β-glucans, while cellular experiments are based on the soluble plus insoluble parts of β-glucans. Beta-glucan preparations were also tested for protein and monosaccharide content. The protein content was determined on a FlashEA 1112 Nitrogen and Protein Analyser (Interscience, Breda, The Netherlands) using the combustion method (Dumas) according to the instructions of the manufacturer. The samples (5–10 mg) were weighted into sample cups and directly measured in duplicate. Methionine (Sigma-Aldrich) was used as standard and cellulose (Sigma–Aldrich) as negative control. The protein content was calculated using 6.25 as nitrogen to protein conversion factor. To determine the monosaccharide composition preparations were hydrolysed with 1 M sulphuric acid at 100 °C for 3 h; only in case of oatβG a pre-hydrolysis step was included with 72% (w/w) sulphuric acid at 30 °C for 1 h. The monosaccharides released were derivatized to alditol acetates and analyzed by gas chromatography using inositol as an internal standard [26]. The absence of uronic acid (UA) was determined with the colorimetric m-hydroxydiphenyl assay [27] using an autoanalyser (Skalar, Breda, The Netherlands) as described before [28]. The β-glucans treated for endotoxin removal (Suppl. Table 1) were also analyzed for molar mass (Suppl. Fig. 1) and protein content prior to this treatment (Suppl. Table 1).

### In vitro polarization and β-glucan treatment of macrophages

Human monocytes were isolated from peripheral blood mononuclear cells (PBMCs) of healthy donors (Sanquin, Nijmegen, The Netherlands) using the quadroMACS system and CD14 microbeads according to the manufacturer’s protocol (Miltenyi Biotec, Leiden, The Netherlands). Monocytes were differentiated into macrophages following 7 days of culture starting at 10^6^ cells/2 ml/well of 24-well plate in RPMI 1640-Glutamax (Gibco) supplemented with 6% human serum (Sanquin, Amsterdam, The Netherlands), 1% antibiotics and 50 ng/ml M-CSF (R&D systems, Minneapolis, MN, USA). Half of the medium was replaced on day 3 and 5 with medium containing 100 ng/ml M-CSF. The cells were considered fully differentiated into M0 macrophages on day 7 and were polarized either with 20 ng/ml TNF-α (R&D systems) and IFNγ (R&D systems) or 20 ng/ml IL-4 (R&D systems) for 18 h. Polarization of both M(TNF-α + IFNγ) and M(IL-4) macrophages was validated by QPCR and detection of typical M1/M2 genes as described by Tang et al. [29]. For experiments, these macrophage subsets were stimulated with medium or 500 μg/ml of β-glucan for 24 h. The MTT 3-(4,5-dimethylthiazol-2-yl)-2,5-diphenyltetrazolium bromide) tetrazolium reduction assay was used to confirm that macrophage cell viability remained within 80–100% range.

### Transcriptome analysis of β-glucan treated macrophages

Total RNA was extracted using TRIzol (Invivogen), and retrieved with the RNeasy mini kit (Qiagen, Venlo, the Netherlands) including RNase-free DNase (Qiagen) treatment for 15 min according to the manufacturer’s instructions. A total amount of 200 ng RNA diluted in milliQ was used for cDNA synthesis in 20 µl total reaction volumes containing 4 µl iScript Reaction Mix and 1 µl Reverse Transcriptase. QPCR amplifications were performed in 20 µl total reaction volumes containing 5 µl cDNA, 10 µl SYBR Green supermix (Bio-Rad) and 2.5 µl forward and reverse primers each. Primers were derived from the Harvard Primerbank (https://pga.mgh.harvard.edu/primerbank/) or designed using Clone Manager Professional 9 and synthesized by Biolegio (Nijmegen, The Netherlands). Primer sequences will be given upon request. Thermal cycling conditions were 95 °C for 90 s, followed by 40 cycles each consisting of 95 °C for 10 s, 58 °C for 10 s and 72 °C for 15 s, and a final elongation step at 72 °C for 2 min, and cycling was performed using the CFX96 Touch Real-Time PCR Detection System (Bio-Rad). Data was analyzed with qBase + software (Biogazelle, Gent, Belgium). Delta Ct values were generated by subtracting the Ct value of beta-actin as an internal standard from the Ct value of the gene of interest, and displayed as the reciprocal of delta Ct. In addition to QPCR, we used the Affymetrix Human Gene 1.1 ST array to study whole genome expression from M(IL-4) macrophages stimulated for 18 h with 500 µg/ml curdlan, yeast-b or zymosan, following which RNA samples were hybridized, washed and scanned according to the manufacturer’s instructions. For analysis, the Benjamini-Hochberg method was used to adjust *p* values, genes were selected that demonstrated a fold change of < − 2 or > 2, and FDR was corrected for *q* < 0.05 (IBMT regularized paired Student’s *t* test). Differential occurrence of pathways was analyzed using IPA 3.0 and activation *z*-scores of > 0.5 (https://www.ingenuity.com).

### Secretion of cytokines and chemo-attractants of β-glucan treated macrophages

Conditioned media of macrophages stimulated for 18 h with β-glucans were tested for the presence of cytokines using human IL-6, IL-10 and TNF-α ELISAs (Biolegend, San Diego, USA) according to the manufacturer's instructions. Conditioned media of M(IL-4) stimulated with curdlan, zymosan or yeast-b were also tested for the presence of chemo-attractants using a Human Chemokine Array kit (ARY017, R&D Systems).

### Patient melanoma samples and analyses of β-glucan treatment

Patient melanoma tissues were freshly collected with informed consent according to institutional guidelines and approved protocols (MEC 2012–436). Tumor tissues comprised cutaneous melanoma stage III or IV and were obtained from various sites (i.e., lymph nodes, in transit metastases, or gut metastases). Tumor tissue was minced into small fragments about 2–3 mm in length and placed in a C-tube (Miltenyi Biotec) with 9 ml RPMI 1640 (Gibco) supplemented with 1% antibiotics. Digestion solution containing 1 ml of 1 mg/ml collagenase A (Sigma-Aldrich) and 100 µl of 1 mg/ml DNAse (Roche, Woerden, The Netherlands) was added, and tumor fragments were subjected to two 30 s mechanical disaggregation steps (program C, GentleMACS), Miltenyi Biotec) each followed by 30 min incubations at 37 ℃. After disaggregation, tumor cell suspensions were passed through a 70 µm strainer, centrifuged for 8 min at 450 × *g*, and washed with 30 ml PBS. Subsequently, single-cell suspensions were reconstituted at 10^6^ cells/ml/well of 24-well plate in RPMI 1640 supplemented with 25 mM HEPES, 200 mM l-glutamine (Invitrogen), 6% human serum (Sanquin, Amsterdam, The Netherlands), 1% antibiotics and 360 U/ml recombinant human interleukin-2 (IL-2) (Proleukin,; Chiron, Amsterdam, The Netherlands), and stimulated with 500 µg/ml curdlan, yeast-b or zymosan. Conditioned media were collected prior to and following 96 h of treatment and were exposed to bead-based multiplex flow cytometry using the pro-inflammatory chemokine LEGENDPlex (BioLegend). In addition, single-cell suspensions, containing tumor-infiltrating myeloid cells and lymphocytes, were subjected to flow cytometry analysis prior to β-glucan treatments. In short, single-cell suspensions were incubated with Human BD Fc Block (BD Pharmingen) for 15 min at RT, followed by staining with the following monoclonal antibodies: CD3-BV21A (SP34-2, BD Biosciences); CD11b-APC (D12, BD Biosciences); CD45-APC-Cy7 (2D1, Biolegend); CD163-PeCy7 (RM311, Biolegend); and 7AAD-PercP (BD Biosciences) for 15 min at RT. Cells were washed once with PBS and subsequently centrifuged for 5 min at 450 g. Finally, cells were re-suspended in 200 μl 1% paraformaldehyde solution. Events were acquired with a BD FACSCelesta and data were processed using FlowJo V10 software (Tree Star Inc., Ashland, OR, USA).

### Statistical analysis

All parameters are presented as means + SEM. Statistical testing was performed with non-paired Student Student’s t test using experimental data that were repeated at least three times using Prism 5 software (Graphad, La Jolla, CA, USA). Regarding the heat map of gene expression **(**Fig. 4) and protein secretion data obtained from Figs. 2 and 3: all data points were first individually standardized, i.e., experimental values were subtracted by mean value and then divided by standard deviation of values of corresponding parameter for corresponding stimulation after which hierarchically clustering was performed in comparison to medium using R studio version 3.5.3. *p* values < 0.05 were considered statistically significant.

## Results

### Physicochemical properties of β-glucan preparations

**Fig. 1:**
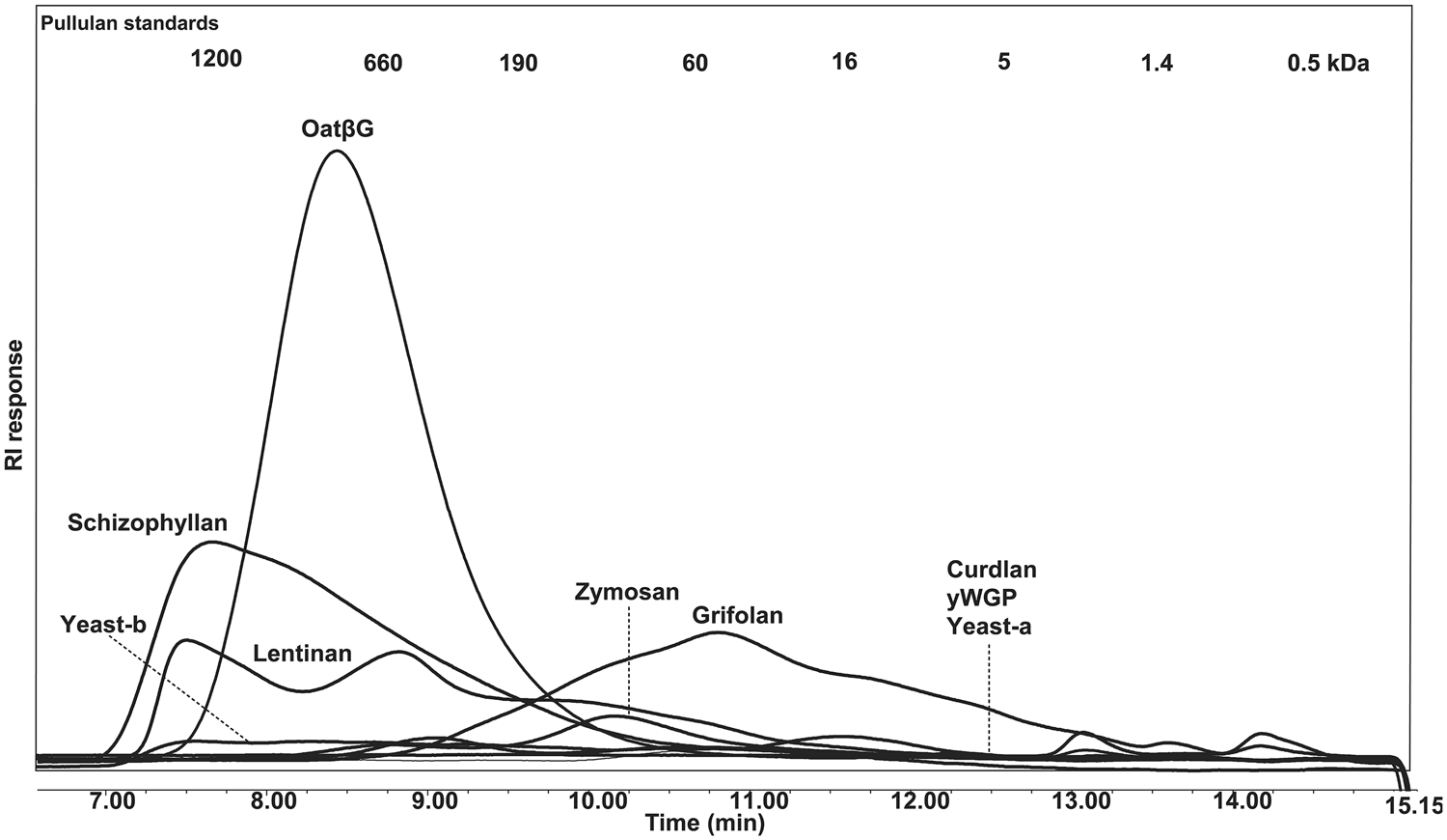
High pressure size exclusion chromatography elution profiles of β-glucan preparations. Chromatograms are displayed of nine β-glucan preparations that were free of LPS and LTA, and used to determine molecular masses and solubility. Pullulan standards were used for calibration and indicated in the top of the figure. The area under the HPSEC curve of oatβG is representing the area of a completely soluble β-glucan

Prior to stimulations of macrophages, all β-glucan preparations were analyzed for presence of bacterial toxins, and those that were contaminated with LPS (i.e. > 0.002 ng/ml) and/or LTA (> 1 µg/ml) were treated to remove these endotoxins and subsequently analyzed for solubility, protein content and sugar composition (see Table 1).With respect to β-glucan solubility, as assessed via areas under the curve in the HPSEC patterns and taken the fact that oatβG has a solubility of 100%, schizophyllan, grifolan and lentinan contain the largest soluble fractions (i.e., 63, 49 and 39%, respectively), whereas curdlan, yeast-a, yeast-b, zymosan and yWGP were barely soluble (< 13%) and could not be analyzed for molecular-mass distribution (Fig. 1). With respect to sugars, all β-glucans were mainly composed of glucose units; besides lentinan, and in line with literature [30, 31], zymosan also contained mannose (11%), whereas lentinan contained both galactose (13%) and mannose (6%). The total % glucan was in all preparations above 50%, except for curdlan and zymosan, and protein content in all preparations was < 20%. Finally, lentinan, schizophyllan and oatβG consist of molecules of a large molar mass (i.e., 60–1200 kDa), while grifolan and zymosan consist of molecules of a smaller molar mass (i.e., 1.4–190 kDa and 16–60 kDa, respectively) (Fig. 1).

**Table 1:** Physicochemical characteristics of β-glucan preparations^a^

β-glucan	Solubility (%)	Protein content (%)	Monosaccharides (mol%)							Total saccharide content (w/w%)	LPS/LTA (ng/ml)/(µg/ml)
			Rha^2^	Ara^2^	Xyl^2^	Man^2^	Gal^2^	Glc^2^	Uronic acid		
OatβG	100	10.8	1	1	0	0	0	96	2	89	0.002/N.D
Curdlan	< 13	9.5	0	0	0	0	0	98	1	38	0.002/N.D
Grifolan	49	19.3	0	1	0	1	2	93	2	72	N.D./N.D
Schizophyllan	63	11.9	0	0	0	0	0	97	2	84	N.D./N.D
Lentinan	39	19.4	0	0	1	6	13	77	3	60	0.002/N.D
yWGP	< 13	16.2	0	1	0	0	0	96	3	64	N.D./N.D
Zymosan	< 13	18.2	1	0	0	11	0	87	2	48	N.D./N.D
Yeast-a	< 13	8	0	0	0	1	0	98	1	56	N.D./N.D
Yeast-b	< 13	11.7	0	0	0	0	0	97	2	75	N.D./N.D

*Rha* rhamnose, *ara* arabinose, *xyl* xylose, *man* mannose, *gal* galactose, *glc* glucose, *LPS* lipopolysaccharide, *N.D.* not detectable

^a^This Table lists molecular masses, monosaccharide content as well as protein content of nine β-glucan preparations that were LPS/LTA free. See Materials and Methods for technical details

### Curdlan, yeast-b and zymosan changed IL-4-polarized macrophages towards an inflammatory phenotype

**Fig. 2:**
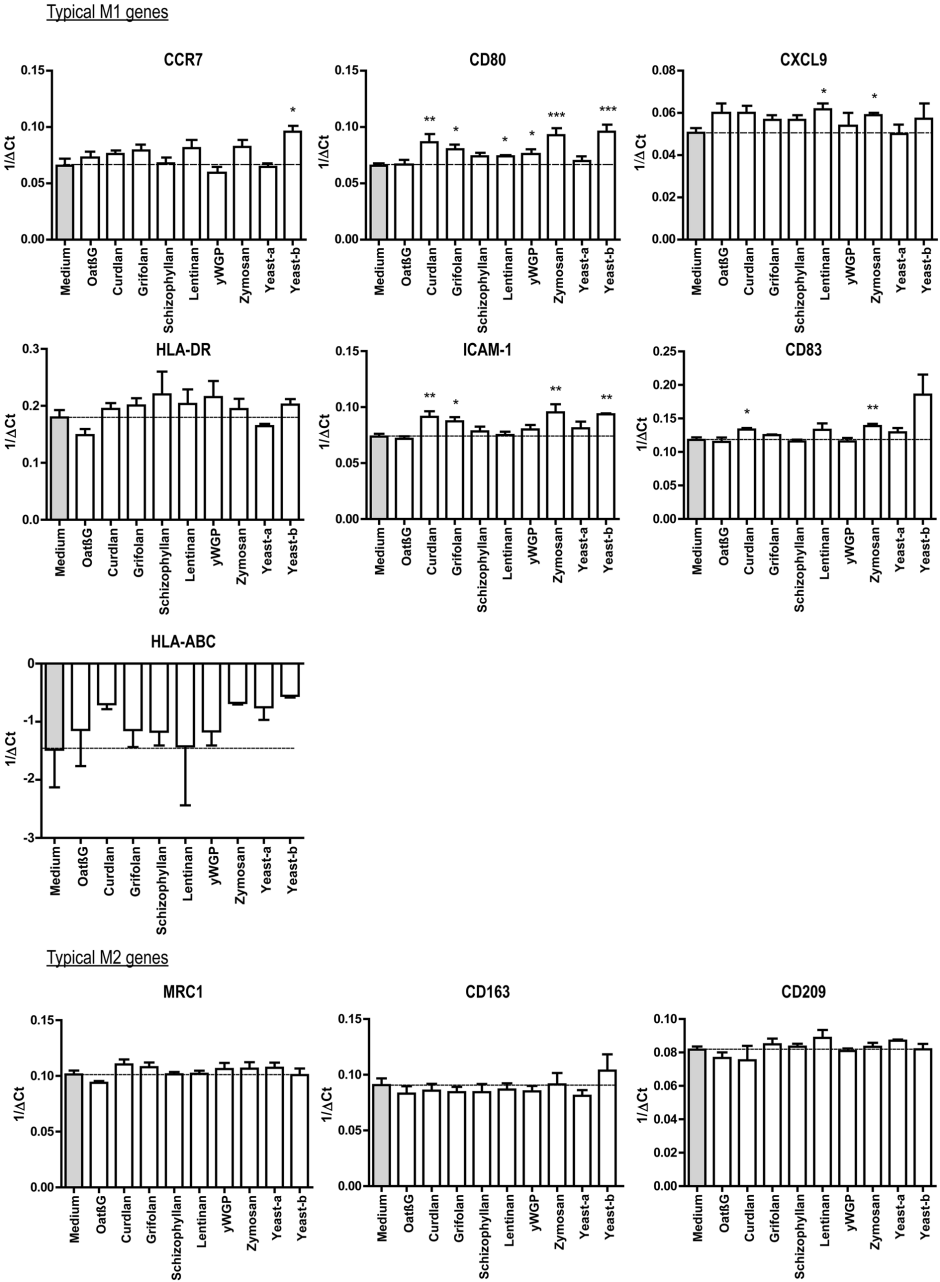
Beta-glucans differentially affect expression of typical M1 and M2 genes in M(IL-4) macrophages. CD14+ monocytes were differentiated into macrophages following 7 days of culture in the presence of M-CSF (see Materials and Methods for details), after which cells were polarized with IL-4 for 18 h. The resulting M(IL-4) macrophages were stimulated for another 18 h with 500 μg/ml curdlan, grifolan, schizophyllan, lentinan, zymosan, yeast-a, yeast-b or 100 µg/ml oatβG or yWGP, and analyzed for gene expression of *CCR7*, *CD80*, *ICAM-1*, *CD83, CXCL9, HLA-ABC, HLA-DR, CD163, CD209* and *MRC1* using QPCR; medium values are used as controls and displayed by gray bars and horizontal lines. Results are shown as average 1/ΔCt (Ct of target gene—Ct of beta-actin) of *n* = 3 different donors, and analyzed with non-paired Student’s *t* test in comparison to medium values. Statistically significant differences: *p < 005, **p < 0.01, ***p < 0.001

**Fig. 3:**
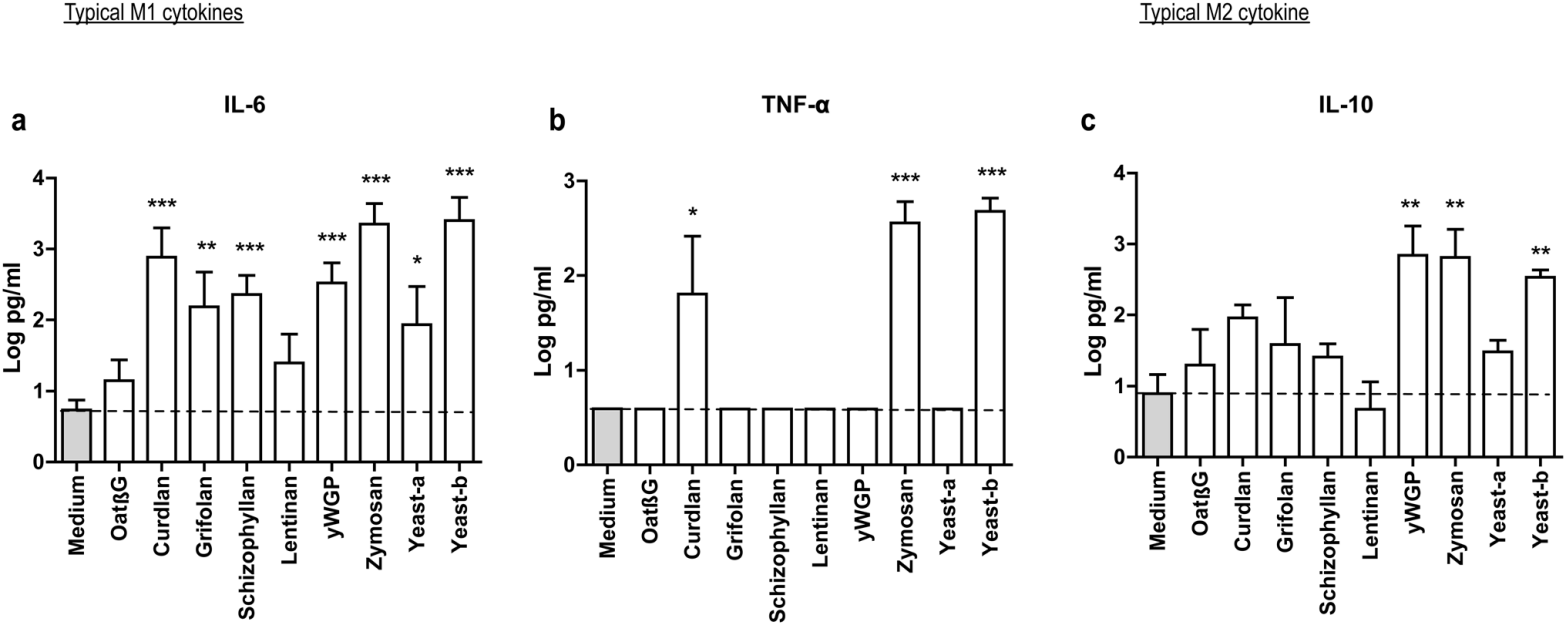
Curdlan, yeast-b and zymosan induce secretion of IL-6, TNF-α and IL-10 by M(IL-4) macrophages. M(IL-4) macrophages were generated and stimulated for 18 h with β-glucans as described in the legend to Fig. 2. Supernatants were collected and tested for the presence of IL-6 (**a**), TNF-α (**b**) and IL-10 (**c**) using ELISAs; medium values are used as controls and displayed by gray bars and horizontal lines. Results are shown as pg/ml cytokine using a logarithmic scale of *n* = 3 different donors, and analyzed with non-paired Student’s *t* test in comparison to medium values. Statistically significant differences: *p < 0.05, **p < 0.01, ***p < 0.001

**Fig. 4:**
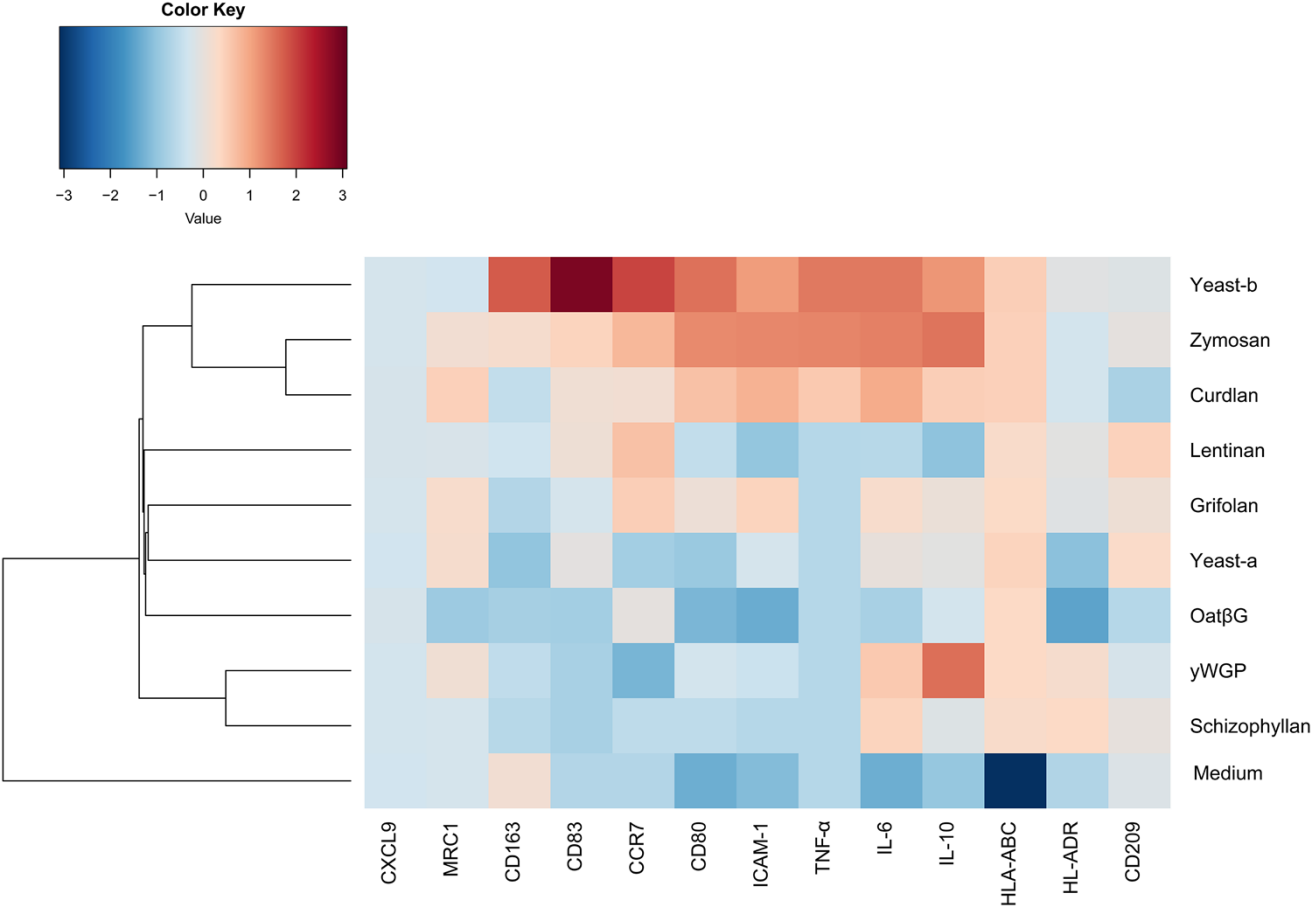
Curdlan, yeast-b and zymosan-stimulated M(IL-4) macrophages show an inflammatory-like state. Heat map of gene expression and protein secretion data obtained from Figs. 2 and 3. All data points were first individually standardized, i.e., experimental values were subtracted by mean value and then divided by standard deviation of values of corresponding parameter for corresponding stimulation. Following standardization, hierarchically clustering was performed

Human monocyte-derived macrophages polarized with IL-4 were exposed to β-glucans and assessed for changes according to expression of genes and secretion of cytokines considered typical for M1 and M2 macrophages. Following exposure to oatβG, schizophyllan or yeast-a no significant alterations in gene expression of the M1 markers *CCR7*, *CD80*, *CXCL9*, *ICAM1*, *CD83*, *HLA-DR*, *HLA-ABC* nor of the M2 markers *CD209, CD163* and *MRC1* were observed (Fig. 2). In contrast, yeast-b and zymosan, curdlan, lentinan, yWGP and grifolan increased gene expression of the M1 markers *CCR7*, *CD80*, *CXCL9*, *ICAM1* and/or *CD83*, but did not affect gene expression of any of the M2 markers. Exposure of non-polarized macrophages to grifolan, curdlan, yeast-b or zymosan demonstrated similar increases in gene expression of *CCR7*, *CD80* and/or *ICAM1*, and gene expressions of these markers became similar to those of polarized non- β-glucan-stimulated M(IFNγ + TNF-α) macrophages (Suppl. Fig. 2a). Again, none of the β-glucans induced significant changes in gene expression of the M2 markers in non-polarized macrophages (data not shown). When analyzing cytokine secretion following β-glucan exposure to M(IL-4) macrophages, we observed that yeast-b and zymosan significantly increased secretion of IL-6, TNF-α and IL-10; curdlan increased secretion of IL-6 and TNF-α; yWGP increased secretion of IL-6 and IL-10; grifolan, schizophyllan and yeast-a significantly increased secretion of IL-6; and none of the stimulations resulted in the secretion of IL-12 (Fig. 3; and data not shown**)**. When testing non-polarized macrophages, we observed very similar cytokine secretions **(**Suppl. Fig. 2b**)**, with the exception of yWGP and zymosan that induced no secretion of IL-6 nor IL-10. Cluster analysis using the combined data of gene expression and cytokine secretion following stimulation of M(IL-4) macrophages revealed that most β-glucans did not strongly change the phenotype of M(IL-4) macrophages **(**Fig. 4**)**. Importantly, curdlan yeast-b, zymosan induced a phenotype dissimilar from M(IL-4) macrophages and closely resembling an inflammatory-like macrophage state.

### IL-4-polarized macrophages treated with curdlan, yeast-b and zymosan showed a unique expression and secretion profile of chemo-attractants

**Fig. 5:**
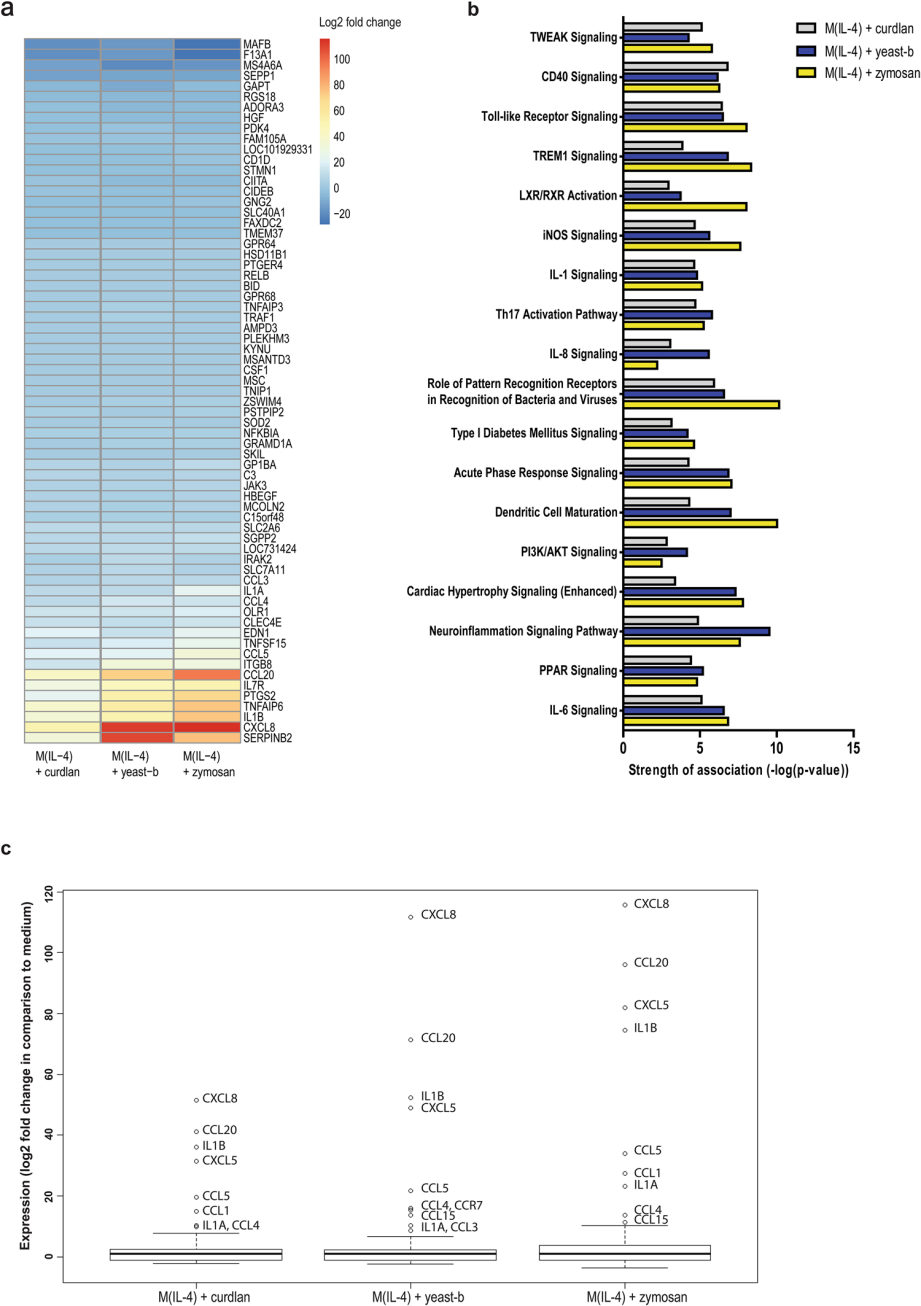
Curdlan, yeast-b or zymosan-stimulated M(IL-4) macrophages demonstrate enhanced gene expression of chemo-attractants and occurrence of immune cell recruitment pathways. M(IL-4) macrophages were generated and stimulated for 18 h with 500 μg/ml curdlan, yeast-b or zymosan as described in legend to Fig. 2, after which whole genome expression analysis was performed using Affymetrix Human Gene 1.1 ST array (see Materials and Methods for details). Genes were selected that demonstrated a fold change < 2 and > 2 when compared to medium, and FDR was corrected for *q* < 0.05 (see Materials and Methods for details). All 67 genes that were differentially expressed following stimulation with either β-glucan when compared to medium were depicted in a heat map (**a**). Canonical pathway activation with a *z*-score > 0.5 according to IPA for any of the β-glucans were depicted in a bar chart (**b**). Differential gene expression of selected chemo-attractants, cytokines and receptors were displayed as fold change compared to medium (**c**)

**Fig. 6:**
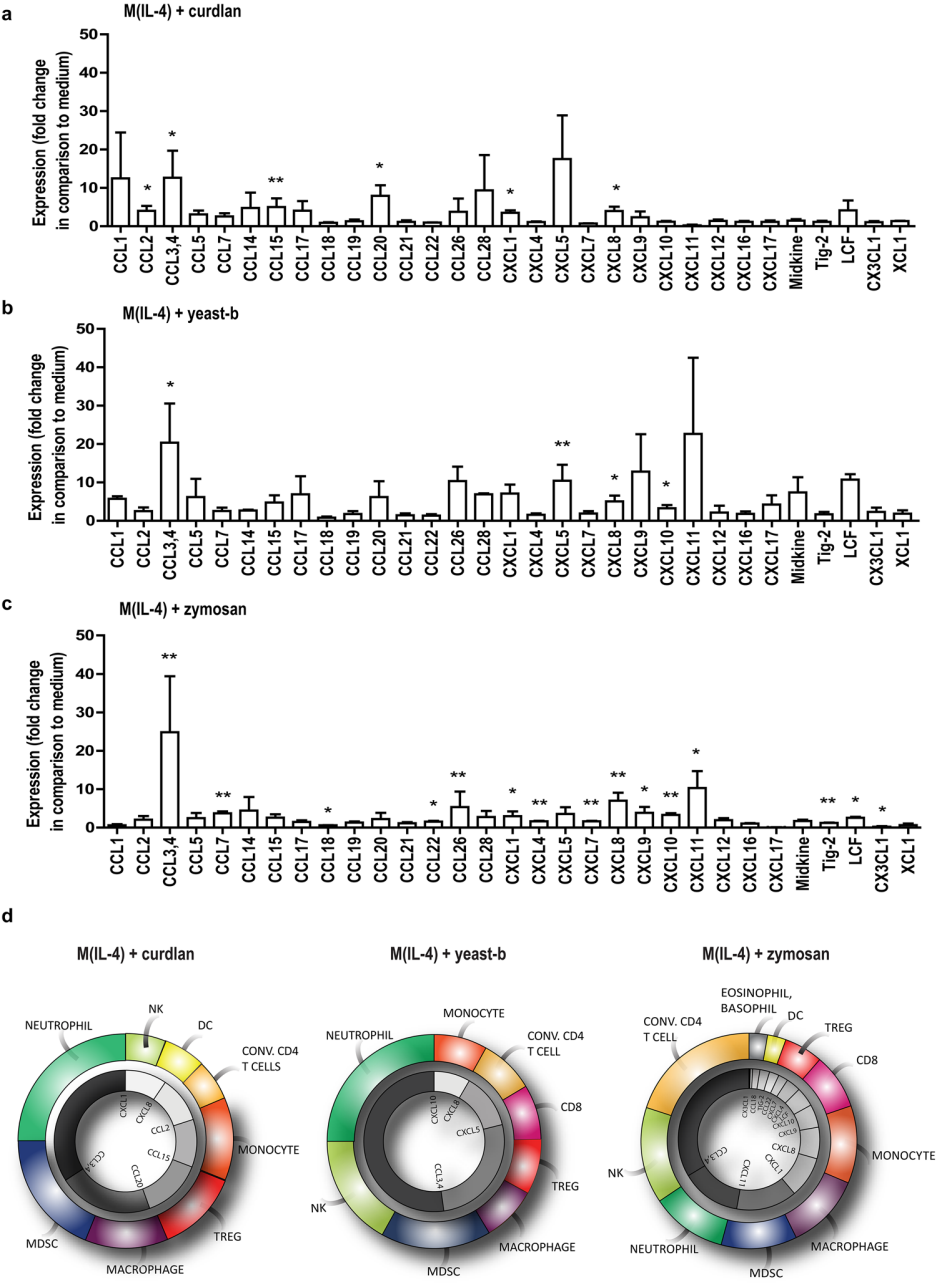
Curdlan, yeast-b or zymosan-stimulated M(IL-4) macrophages show unique chemoattractant secretion profiles. M(IL-4) macrophages were generated and stimulated for 18 h with 500 μg/ml curdlan (**a**), yeast-b (**b**), or zymosan (**c**) as described in legend to Fig. 2. Supernatants were collected (*n* = 3 independent experiments) and tested for the presence of 31 chemo-attractants using a Human Chemokine Array kit (see Materials and Methods for details). Expression is displayed as fold change in comparison to medium using a logarithmic scale, and tested with non-paired Student’s *t* test. Pie charts show the induced potential of recruitment of immune cells based on the secretion profiles of chemo-attractants per β-glucan (**d**). Statistically significant differences: *p < 0.05, **p < 0.01

To better understand the change towards an inflammatory state induced in M(IL-4) by exposure to curdlan, yeast-b or zymosan we set out to perform whole genome expression analysis. To this end, we stimulated M(IL-4) with these three β-glucan preparations. Our results showed that these three β-glucans significantly affected expression of in total 501 genes (fold change < − 2 or > 2 and *q* < 0.05) when compared to medium stimulation. Venn analysis of this set of genes showed that differential expression of 4, 259 and 50 genes were unique to curdlan, yeast-b and zymosan, respectively (Suppl. Fig. 3**)**. Furthermore, 67 genes were differentially expressed following stimulations with either of the three β-glucans (Suppl. Figs. 3 and 5a). It is noteworthy that gene expression of inflammatory cytokines and chemo-attractants, such as *CXCL8*, *CCL20*, *TNFSF15* and *IL1B,* was increased. These changes in gene expressions were most pronounced following stimulation with zymosan. Ingenuity Pathway Analysis (IPA) identified canonical pathways that best captured these differentially expressed gene sets (Fig. 5b**).** These pathways relate mostly to immune cell recruitment (i.e., CXCL8 signaling, IL-1 signaling) as well as activation of myeloid cells and lymphocytes (i.e., CD40 signaling, Toll-like receptor (TLR) signaling, and TREM1 signaling). We zoomed in on the increased gene expression of chemo-attractants and cytokines upon exposure of M(IL-4) to curdlan, yeast-b or zymosan, which is shown in Fig. 5c. To verify secretion of chemo-attractants, we analyzed 31 chemo-attractants using a protein array and found that curdlan resulted in increased secretion of CCL2, CCL3/CCL4, CCL15, CCL20, CXCL1, and CXCL8; yeast-b of CCL3/CCL4, CXCL5, CXCL8, and CXCL10; and zymosan of CCL3/CCL4, CCL7, CCL22, CCL26, CXCL1, CXCL4, CXCL7, CXCL8, CXCL9, CXCL10, CXCL11, TIG-2, and LCF (Fig. 6a–c**)**. The above chemoattractant production profiles enabled identification of potential immune cell populations that would be recruited as a consequence of M2 macrophages stimulated with these β-glucans [32–34] **(**displayed as pie charts, Fig. 6d**)**.

### Curdlan, yeast-b and zymosan enhanced production of chemo-attractants by freshly isolated melanoma-associated macrophages

**Fig. 7:**
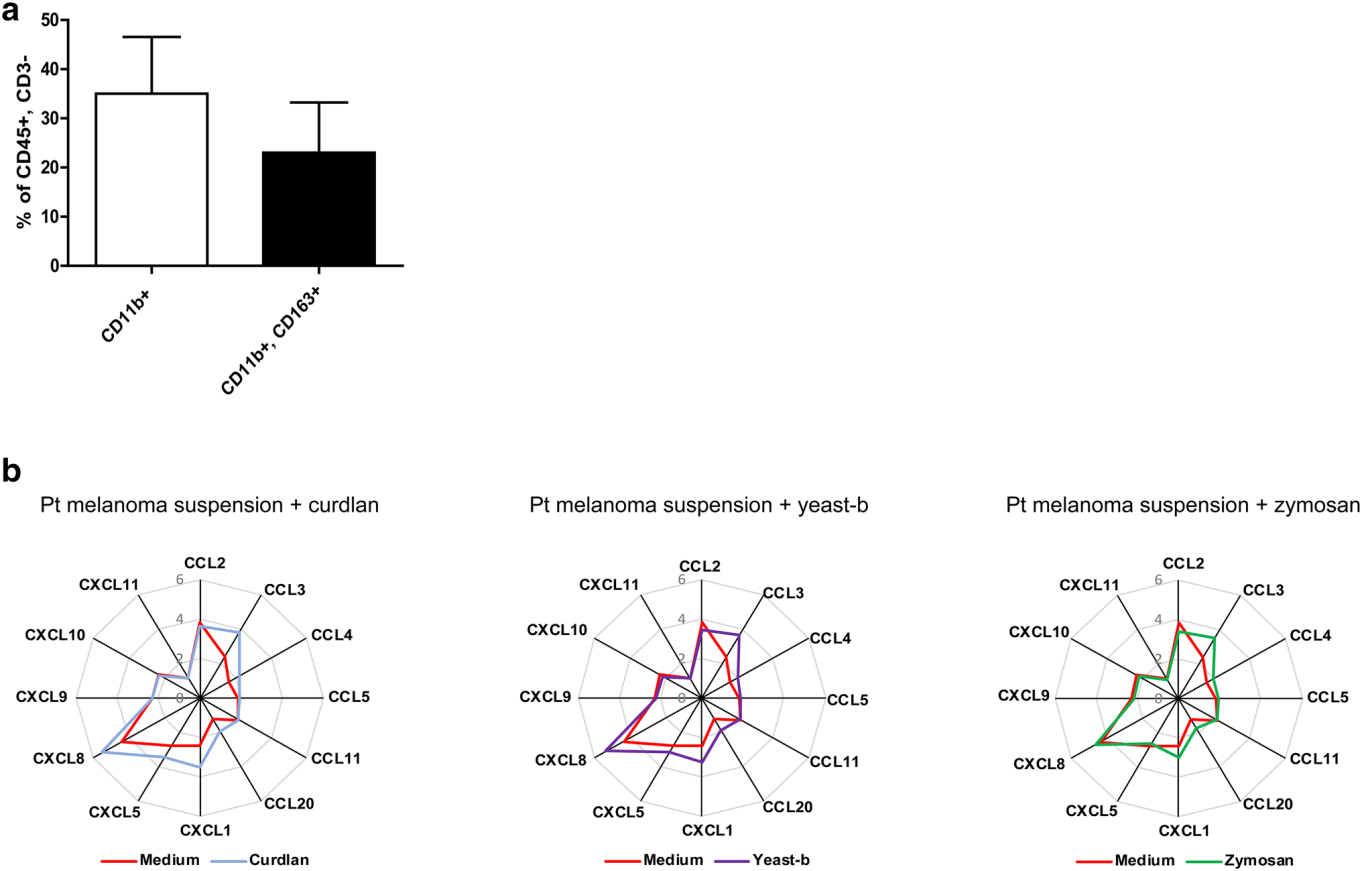
Curdlan, yeast-b and zymosan enhance chemoattractant secretion by patient-derived melanoma cell suspensions. Melanoma tissues were processed into single-cell suspensions and subjected to flow cytometry analysis. Total leucocytes were first gated on a side scatter (SSC)/7AAD, and then gated for CD45 + , CD3− cells (~ 10%). These cells were further gated for CD11b^+^ (myeloid cells) and CD11b^+^, CD163^+^ (TAMs) which population frequencies were expressed as percentage of CD45 + cells and CD3 − cells (**a**). These patient-derived melanoma single-cell suspensions were stimulated for 24 h with 500 μg/ml curdlan, yeast-b, or zymosan, after which supernatants were collected and tested for the presence of chemo-attractants using a Human Chemokine Array kit. Secretion of a chemo-attractants is displayed as fold change in comparison to medium using spin diagrams. Values represent logarithmic averages after transformation of measured values in pg/ml of *n* = 3 different donors (**b**). Statistical differences were tested with a paired Student’s *t* test (*p* > 0.01)

To analyze whether enhanced chemoattractant production is also occurring when using patient-derived TAMs, curdlan, yeast-b and zymosan were used to stimulate single-cell populations from freshly collected patient melanoma biopsies. Flow cytometry revealed that on average 15% of single-cell suspensions expressed CD45 and were CD3 negative, and of these CD45 + /CD3− cells about 35% expressed CD11b and about 20% expressed both CD11b and CD163 **(**Fig. 7a**)**. Single-cell suspensions were treated with 500 μg/ml of β-glucans for 24 h revealed that all three β-glucans enhanced secretion of CCL3, CCL4, CCL20, CXCL1 and CXCL8, whereas yeast-b and curdlan also enhanced secretion of CXCL5 (Fig. 7b).

## Discussion

In this study, we report on a novel feature of β-glucans, namely to reprogram IL-4-polarized macrophages and TAMs and changing them into macrophages that uniquely produce immune-potentiating chemo-attractants. Starting with nine different bacteria-, fungi-, yeast- or cereal-derived β-glucans, we first excluded the presence of LPS and LTA, after which we measured molar mass, solubility, protein and saccharide contents. This is not customary for many β-glucan-based studies [2], although in our view of critical importance to value the potential neo-adjuvant effect of these compounds, and better understand structural features that contribute to biological activity. Physicochemical analysis revealed that curdlan, yeast-b and zymosan preparations all contained high fractions of insoluble material (> 87%). The observed relationship between biological activity and insolubility of β-glucans is in accordance with other studies, which observed immune stimulatory effects particularly of branched [35] and insoluble β-glucans [36–38]. Insoluble β-glucan can be phagocytosed by dendritic cells and macrophages via the dectin-1 receptor pathway [39]. This pathway is considered essential for the activation of these innate immune cells, which in turn results in a T-cell response. Fragmented and/or soluble β-glucans, however, may not unequivocally bind nor stimulate the same receptors as full length β-glucans. small β-glucan particles with a backbone length below seven glucose units cannot bind to dectin-1, and soluble β-glucans cannot initiate clustering of dectin-1 in immunological synapses, and consequently cannot activate this receptor [39]. It is noteworthy, however, that soluble β-glucans are able to bind to the complement receptor 3 and support humoral immune responses [40, 41]. When taken current viewpoints together, both soluble and insoluble β-glucan fractions are capable of supporting immune responses, yet we argue that the β-glucan activity towards the production of chemo-attractants and potentially towards a cellular immune response is expected to be linked to insoluble β-glucans. Besides solubility, we cannot exclude that other physicochemical properties of β-glucans also contribute to the observed effects, such as degree of branching and higher order structures [42]. The same holds true for differential expression levels of PRRs upon exposure to β-glucans, as we have previously observed for dietary fibers and again in our microarray data ([29] and data not shown). In short, we advocate further systematic studies into structure–function relationships using β-glucans as well as PRR usage to better understand the exact mechanism of actions of β-glucans.

Our β-glucan preparations were employed to a human monocyte-derived macrophage culture system providing non-polarized macrophages and M(IL-4) macrophages and demonstrated increases in gene expression of the co-stimulatory molecule CD80 by lentinan, yWGP, yeast-b, curdlan, zymosan and grifolan, of which the latter four also significantly increased the expression of another M1 marker, namely the adhesion molecule ICAM1. In contrast, none of the β-glucans reduced expression of the M2 markers CD209, CD163 and MRC1, suggesting that β-glucans are able change the M(IL-4) macrophage phenotype, but not completely revert this phenotype (Fig. 2). This is in line with a report demonstrating the potential of IMPRIME to reduce the expression level of CD163 on the surface of macrophages and enhance macrophage-induced T-cell proliferation and IFNγ secretion [43]. Curdlan, yeast-b and zymosan were the only β-glucans that enhanced secretion of IL-6 and TNF-α, whereas yeast-b and zymosan enhanced secretion of IL-10 by M(IL-4) macrophages (Fig. 3). Expression of IL-10 may depend on the level of glycolytic commitment of macrophages [44]. M1 macrophages generally use glycolysis, while oxidation is generally used by M2 macrophages as their main glucose metabolic pathway [45]. these metabolic pathways and their downstream products determine the biologic activity of macrophages [46]. In tumors, the metabolic activity of TAMs is in part guided by oxygen tension, such that TAMs that accumulate in a normoxic tumor area adopt a more M1-like phenotype, whereas those that accumulate in a hypoxic tumor adopt a more M2-like phenotype [47]. Interestingly, IL-10 acts as feedback mechanism to limit glycolytic commitment and to balance the inflammatory phenotype of macrophages, and its expression may have been preceded by an inflammatory state of macrophages. Absence of IL-10 production by non-stimulated macrophages, whether they be non-polarized, M(IL-4) or M(TNFα + IFNγ) (Fig. 3 and Suppl. Fig. 2b), suggests that IL-10 expression is a consequence of β-glucan-ligation of pattern recognition receptors [48]. Unexpectedly, there was no detectable IL-12 within the supernatant of β-glucan-stimulated macrophages. This might be partly explained by the production of TNF-α, which is a potent inhibitor of IL-12 secretion by human macrophages [49]. The co-ligation of dectin-1 with several different TLRs has been demonstrated to up-regulate TNF-α secretion [50], but also to down-regulate IL-12 secretion [51], which may imply that β-glucans may mediate the observed effects (in part) through TLRs. Integrating data obtained from QPCRs and ELISAs into a heatmap demonstrated that curdlan, yeast-b and zymosan induced the most pronounced skewing away from M(IL-4) harboring an inflammatory-like state (Fig. 4).

The impact of curdlan, yeast-b and zymosan β-glucan on M(IL-4) macrophages was further assessed using whole genome expression analysis. When comparing the three β-glucan stimulations for differentially expressed genes, we observed that a set of 67 genes was shared (Fig. 5a**)** and 4, 259 and 50 genes were unique for curdlan, yeast-b and zymosan, respectively (Suppl. Fig. 3). When looking at shared genes, we observed that expression of F13A1 and MS4A6A, considered typical for human IL-4-polarized macrophages [52], were reduced, whereas expression of PTGS2, TNFAIP6, SERPINB2, ITGB8, typical for inflammation, were increased compared to non-stimulated M(IL-4) macrophages. The occurrence of the iNOS, IL-1, IL-6 and CXCL8 signaling pathways in M(IL-4) macrophages stimulated with these β-glucans is also in line with an inflammatory state (Fig. 5b). Interestingly, low-dose irradiation, which programs the differentiation of iNOS-positive M1 macrophages and induces expression of chemo-attractants, causes efficient recruitment of tumor-specific CD8 T cells in pancreatic carcinoma [53]. Other affected pathways involved pattern recognition receptors and were illustrated by Toll-like receptor and TREM1 signaling pathways. Collectively, gene and pathway analyses revealed that curdlan, yeast-b and zymosan evoked concerted increases in expression of chemo-attractants (Fig. 5c). This was confirmed by protein analysis, which enabled the identification of unique production profiles for the different β-glucans (Fig. 6a–c). The induction of CCL3, CCL4 and CXCL8 in M(IL-4) macrophages was a similar feature between all three β-glucans, with CCL3 and 4 (MIP1 alpha and beta, respectively) and CXCL8 (IL-8) being chemokines that are mainly involved in the recruitment and activation of monocytes and neutrophils [32–34]. Both curdlan and zymosan enhanced secretion of CCL2; mainly produced by monocytes and macrophages in response to *e.g.* growth factors, oxidative stress or other pro-inflammatory cytokines. CCL2 recruits circulating monocytes and macrophages to the site of injury or inflamed tissue [54]. The chemokines CXCL9 and CXCL10, secreted upon exposure to zymosan or yeast-b and zymosan, respectively, are produced by monocytes, macrophages and cancer cells in response to IFN-y [55]. Both chemokines attract activated T lymphocytes and are required for anti-tumor immune responses following immune checkpoint blockade when produced by macrophages [56].

Literature-based analyses of the significantly enhanced chemokines showed that yeast-b induced the potential to recruit neutrophils and NK cells; curdlan induced the potential to recruit MDSC and neutrophils; and zymosan induced the potential to recruit the most diverse panel of immune cell types, such as conventional CD4 T cells, eosinophils, basophils and monocytes (Fig. 6d). Interestingly, production of CCL18 and CX3CL1 by M(IL-4) macrophages was decreased following stimulation with zymosan. This finding corroborates the potential anti-tumor effects of zymosan-stimulated M(IL-4) macrophages since CCL18 profoundly influences macrophage maturation and induces an M2-like phenotype [57], whereas CX3CL1 is known to control the recruitment of TAMs to tumor sites [58].

When extending findings with M(IL-4) polarized macrophages to patient melanoma-derived TAMs (which we defined as CD45 + CD3-CD11b + CD163 + cells), we observed again that these three β-glucans enhanced secretion of chemo-attractants (Fig. 7). Notably, the induction of CCL3, CCL4 and CXCL8 was again a similar feature between all three β-glucans. Concentrations of β-glucans used in our experiments may over represent actual peripheral concentrations following oral consumption, and accessibility of macrophages was maximized using tumor-derived single-cell suspensions. Despite these technical challenges, our data argue that β-glucans shift CD11b, CD163-positive intra-tumoral myeloid cells into myeloid cells that secrete chemo-attractants. Future studies are required to validate β-glucans’ effects towards the secretion of chemo-attractants by TAMs and to assess their support towards recruitment and anti-tumor responses of immune effector cells. Along these lines, we are currently testing the potential of β-glucans as neo-adjuvant through oral delivery in support of adoptive T-cell therapy to treat melanoma in a preclinical model. A number of clinical phase I and II studies in which β-glucans are used to support various therapies for multiple types of tumors, are already ongoing and further substantiate the expectancy of these compounds in combination therapies in the oncology setting (clinicaltrials.gov).

### Electronic supplementary material

Below is the link to the electronic supplementary material.

Supplementary file1 (PDF 463 kb)

## Abbreviations

CR3: Complement receptor 3
Conv CD4 T cell: Conventional cluster of differentiation 4 T cell
LacCer: Lactosylceramide
LCF: Lymphocyte chemoattractant factor
LTA: Lipoteichoic acid
Tig-2: Tazarotene-induced gene 2 protein
Treg: Regulatory T cell
yWGP: Yeast whole glucan particle
