# Supplementary material for: Curdlan, zymosan and a yeast-derived β-glucan reshape tumor-associated macrophages into producers of inflammatory chemo-attractants
Source: Cancer Immunol Immunother. 2020 Aug 29;70(2):547–61. doi: 10.1007/s00262-020-02707-4 (PMC7889676; doi:10.1007/s00262-020-02707-4)
Supplement: Supplementary file 1 — Supplementary file1 (PDF 463 kb) [file 262_2020_2707_MOESM1_ESM.pdf]

Supplementary figures and table

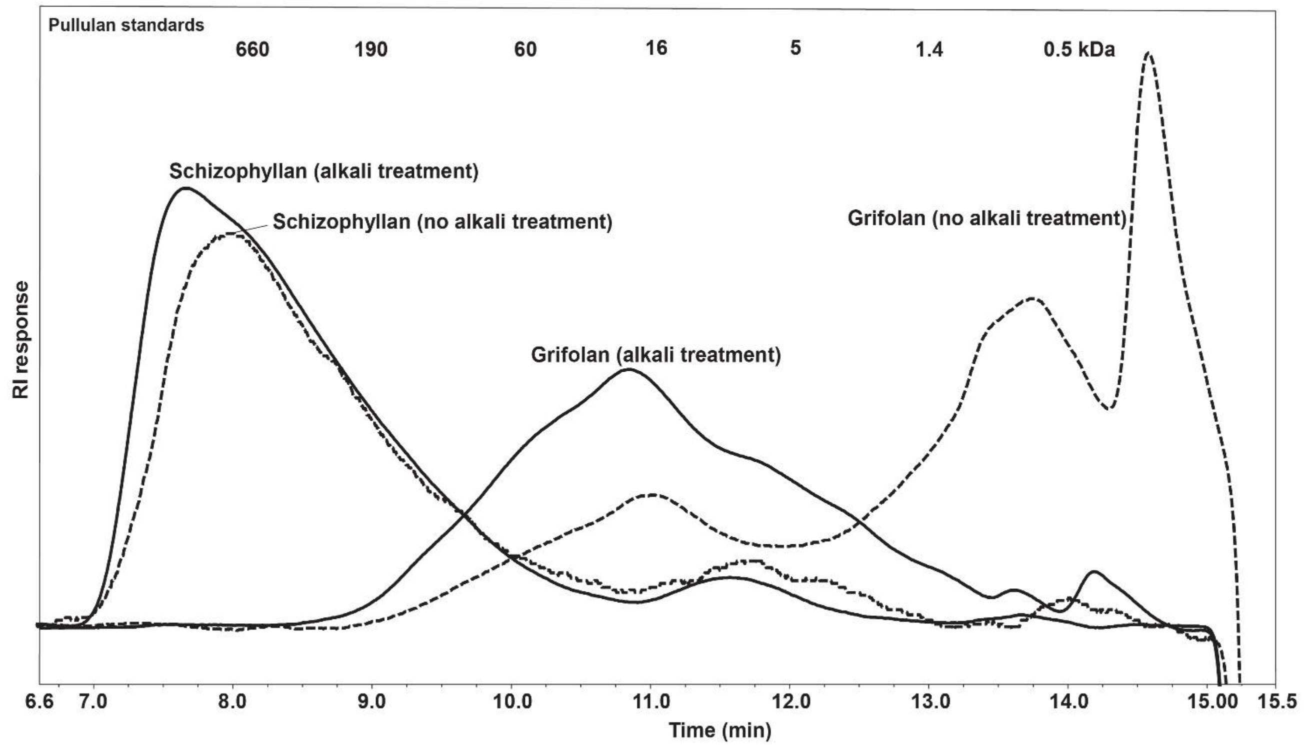

sFig. 1 High pressure size exclusion chromatography elution profiles of β-glucan preparations before and after alkali treatment. Chromatograms are displayed of two β-glucan preparations, schizophyllan and grifolan, pre- and post-treatment for alkali. Pullulan standards were used for calibration and indicate in the top of the figure.

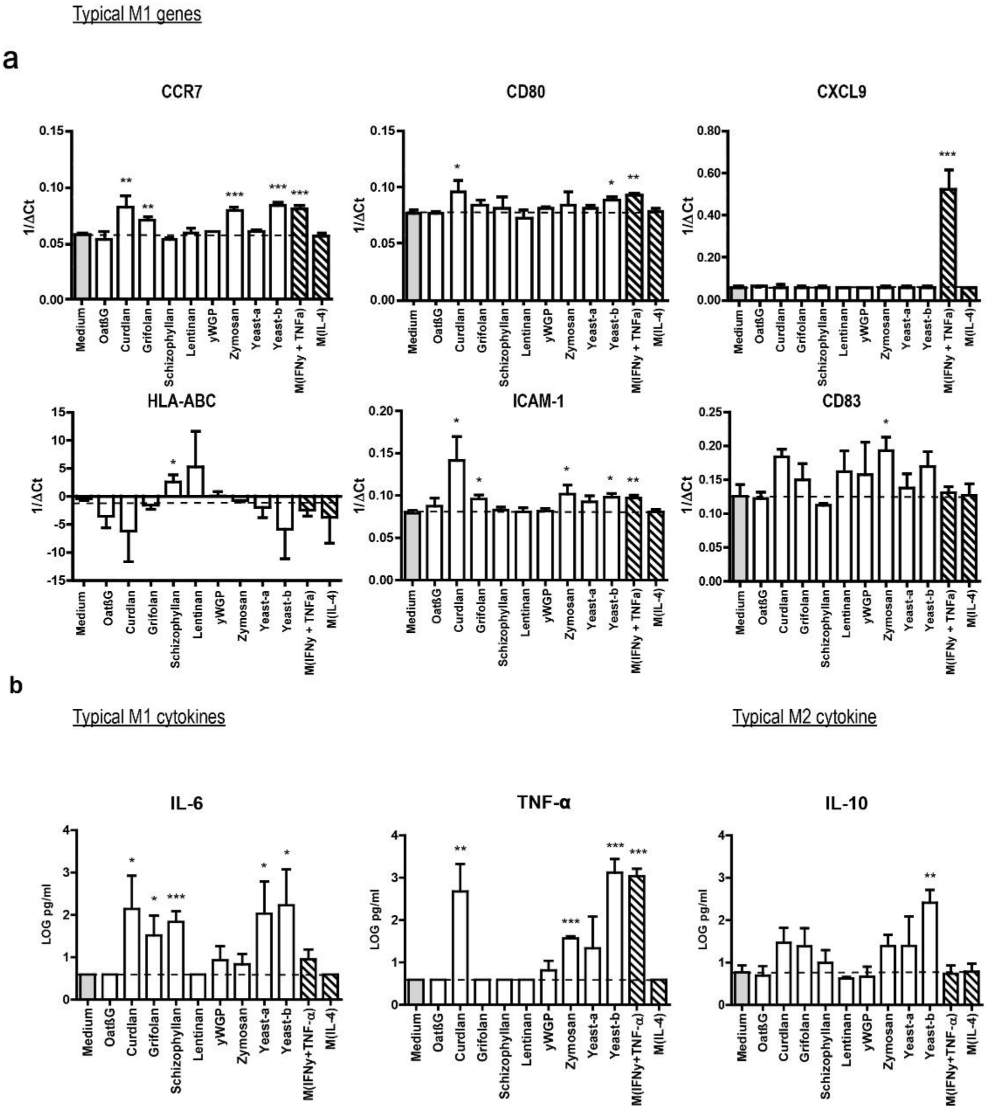

sFig. 2 Beta-glucans differentially affect expression of typical M1 and M2 markers in non-polarized macrophages. CD14+ monocytes were differentiated into macrophages following 7 days of culture in the presence of M-CSF (see Materials and Methods for details). The resulting macrophages were polarised for 18h with 500 µg/ml curdlan, grifolan, schizophyllan, lentinan, zymosan, yeast-a or yeast-b, or 100 µg/ml oatβG or yWGP. In control settings, macrophages were polarised for 18h either with TNF-α and IFNγ or IL-4 to generate M(TNF-α+IFNγ) or M(IL-4) macrophages, respectively. Following stimulation or polarization, macrophages were analyzed for gene expression of *CCR7, CD80, CXCL9, HLA-ABC, ICAM-1* and *CD83* using QPCR; results are shown as average 1/ΔCt (Ct of target gene - Ct of beta-actin) of n=3 different donors (a). Supernatants were collected and tested for the presence of IL-6, TNF-α and IL-10 using ELISAs; results are shown as pg/ml cytokine using a logarithmic scale (b). In both (a) and (b) medium values are used as negative controls and displayed by gray bars and horizontal lines, and values of polarized macrophages are used as positive controls and displayed by shaded bars (at right-end of x-axes). Data is shown of n=3 different donors, and differences were analysed by a non-paired Student's t-test. Statistically significant differences: *p<0.05, **p<0.01, ***p<0.001.

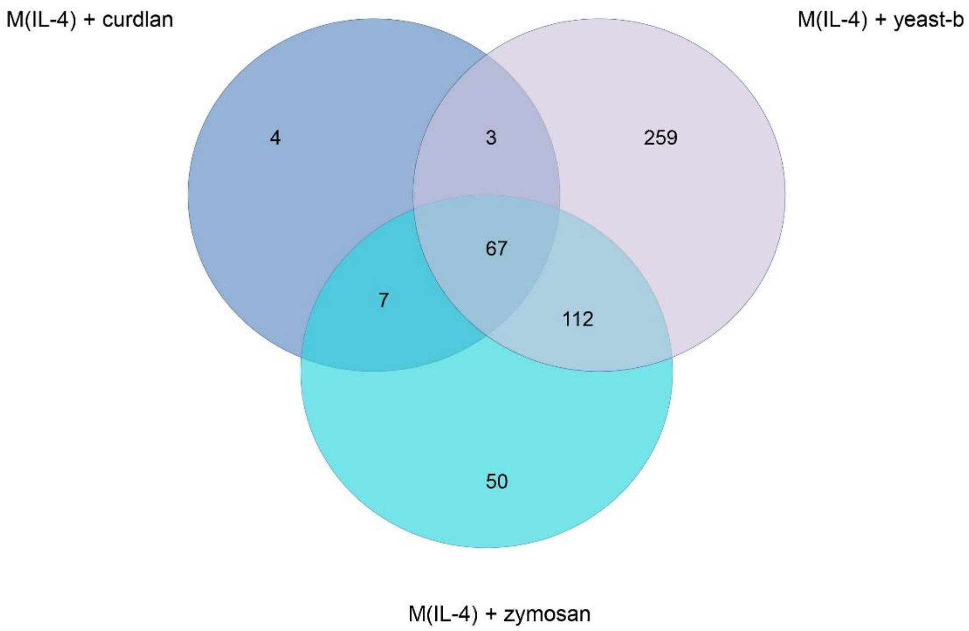

sFig. 3 Shared and non-shared genes that are differentially expressed by M(IL-4) macrophages following exposure to curdlan, yeast-b and zymosan. M(IL-4) macrophages were generated and stimulated for 18h with 500 μg/ml curdlan, yeast-b or zymosan as described in legend to Fig. 2, after which whole genome expression analysis was performed as described in the legend to Fig. 5. Differentially expressed genes described in the legend in Fig. 5 are illustrated in a Venn diagram showing overlap and non-overlap between those genes expressed following stimulations with the different β-glucans. The differentially expressed genes shared among the three β-glucan stimulations are displayed in Fig. 5a.

sTable 1. Physicochemical characteristics of β-glucan preparations before alkali treatment.

β-glucan	LPS content (ng/ml)	LTA content (μg/ml)	Protein content (%)
Grifolan	4.41	N.D.	36.7
Schizophyllan	16.3	N.D.	14.7
Zymosan	3.54	N.D.	28.6
Yeast-a	0.002	2	8
Yeast-b	25.76	N.D.	16.4
